# Transcriptome Analysis Revealed the Mechanism of Inhibition of Saprophytic Growth of *Sparassis latifolia* by Excessive Oxalic Acid

**DOI:** 10.3390/cells11223636

**Published:** 2022-11-16

**Authors:** Zhiheng Qiu, Xinyi Wang, Shuang Wang, Nuo Cai, Jing Huang, Miaoyue Wang, Lili Shu, Tianlai Li

**Affiliations:** 1 College of Horticulture, Shenyang Agricultural University, Shenyang 110866, China; 2 Key Laboratory of Protected Horticulture of Education Ministry and Liaoning Province, Shenyang 110866, China

**Keywords:** *Sparassis latifolia*, oxalic acid, slow mycelial growth, transcriptome analysis, saprophytic growth

## Abstract

*Sparassis latifolia*, a highly valued edible fungus, is a crucial medicinal and food resource owing to its rich active ingredients and pharmacological effects. Excessive oxalic acid secreted on a pine-sawdust-dominated substrate inhibits its mycelial growth, and severely restricts the wider development of its cultivation. However, the mechanism underlying the relationship between oxalic acid and slow mycelial growth remains unclear. The present study reported the transcriptome-based response of *S. latifolia* induced by different oxalic acid concentrations. In total, 9206 differentially expressed genes were identified through comparisons of three groups; 4587 genes were down-regulated and 5109 were up-regulated. Transcriptome analysis revealed that excessive oxalic acid mainly down-regulates the expression of genes related to carbohydrate utilization pathways, energy metabolism, amino acid metabolism, protein synthesis metabolism, glycan biosynthesis, and signal transduction pathways. Moreover, genes encoding for wood-degrading enzymes were predominantly down-regulated in the mycelia treated with excessive oxalic acid. Taken together, the study results provide a speculative mechanism underlying the inhibition of saprophytic growth by excessive oxalic acid and a foundation for further research on the growth of *S. latifolia* mycelia.

## 1. Introduction

*Sparassis latifolia* is a major commercial mushroom widely cultivated and consumed in Asia [1]. It is a good edible and medicinal dietary food composed of rich flavor substances and bioactive components [2]. A substrate containing pine sawdust as the main ingredient is the most commonly used raw material for its commercial cultivation, but its slow mycelial growth rate severely restricts the expansion of its cultivation scale [3]. However, few studies have investigated the reasons for the slow mycelial growth of *S. latifolia*.

The utilization of a substrate by *S. latifolia* is the key to directly understanding its mycelial growth. Unlike most saprophytic edible fungi, *S. latifolia* possesses two different nutritional modes: saprophytic and parasitic [4]. In previous studies, we found that its mycelium secretes a large amount of oxalic acid during its growth, which can inhibit mycelial growth [5]. Nevertheless, the mechanism underlying the growth rate of *S. latifolia* mycelia to oxalic acid during the process of saprophytic growth has not been deeply elucidated.

Oxalic acid is an important pathogenic or toxic factor in the interaction between pathogenic fungi and their hosts. This organic acid can attack defense substances on the plant surface, making it easier for pathogens to infect plants. Oxalic acid accumulates continuously in the infected site, directly inducing toxin action and leading to host cell necrosis [6,7]. Oxalic acid is also a good electron donor or electron acceptor. It can not only be used as a metal chelator in the process of wood degradation catalyzed by manganese peroxidase, but also as an osmotic pressure and pH regulator [8]. Moreover, oxalic acid secreted by parasitic fungi offers a favorable acidic environment, thereby increasing the activity of wood-degrading enzymes (laccase, peroxidase, xylanase, etc.) secreted by these fungi and facilitating mycelial invasion of the host plant tissue [9]. Many wood-rotting fungi can also secrete oxalic acid to assist wood degradation during the saprophytic process, and its concentration is actively regulated by fungi [10]. The participation of the Feton reaction is vital for the degradation of pine sawdust, and oxalic acid concentration has a bidirectional regulatory effect on the Feton reaction, which means a low concentration promotes the reaction and a high concentration inhibits it [11]. Therefore, oxalic acid is a crucial player in pine sawdust degradation during the saprophytic process of *S. latifolia*.

Gene expression profiles of edible fungi have recently been considered effective tools for understanding molecular mechanisms. RNA-seq is a practicable method for determining differential transcript levels of edible fungi under different treatments [12]. Transcriptome-based analysis of a wood-rot fungus *Abortiporus biennis* revealed that the addition of oxalic acid significantly promoted the expression of cellulolytic enzyme genes, but significantly inhibited the expression of lignolytic enzyme genes [13]. Xing et al. used transcriptomic profiling to identify candidate genes involved in sclerotial formation by *Polyporus umbellatus* in the presence of oxalic acid [14]. These transcriptome data indicated that RNA-seq is a rapid and high-throughput approach for obtaining comprehensive differentially expressed genes (DEGs) of oxalic-acid-treated edible fungi.

No researchers have currently focused on the reason for the slow saprophytic growth rate of *S. latifolia* and the inhibitory role of oxalic acid in its slow mycelial growth rate. Illumina sequencing technology was used here to detect the differential properties of *S. latifolia* at different oxalic acid concentrations and thus establish a critical basis for elucidating the mechanism underpinning the slow mycelial growth rate during the saprophytic process.

## 2. Materials and Methods

### 2.1. Strain, Sample Collection, and Preparation

*S. latifolia* CCMJ 1100 was obtained from the Culture Collection Center of Mycology of Jilin Agriculture University. For the pure culture of *S. latifolia*, a mycelial disc (diameter: 5 mm) was cultured on the center of potato dextrose peptone agar (PDPA) at 24 °C [15]. To grow mycelia, 10 mycelial discs (5 mm) of *S. latifolia* were inoculated into pine sawdust medium (PSM, 76% pine sawdust, 18% bran, 2% corn flour, 1.5% sucrose, 1.5% gypsum, and 1% calcium superphosphate) in a bag (17 × 35 cm) and incubated at 24 °C [5]. To identify the expression profiles of target genes affected by excessive oxalic acid, the mycelia were collected from PSM (CK), PSM with 10 mM oxalic acid (OA), and 4 mM 3,3-difluorooxaloacetate (IOA, an inhibitor of oxalic acid synthesis) after 20 days of incubation at 24 °C, mixed thoroughly, frozen immediately in liquid nitrogen, and stored at −80 °C for RNA extraction. A 3,3-Difluorooxaloacetate (concentration: 98%) was prepared according to a previously described method [16].

### 2.2. RNA Isolation

Total RNA was isolated using the Trizol Reagent (InvitrogenLife Technologies, Carlsbad, CA, USA) according to the manufacturer’s protocol. The Nanodrop 2000 spectrophotometer (Thermo Fisher Scientific, Waltham, MA, USA) and the Agilent 2100 Bioanalyzer and 2100 RNA nano 6000 assay kit (Agilent Technologies, Santa Clara, CA, USA) were used to evaluate the concentration and purity and the integrity of RNA samples, respectively.

### 2.3. Library Construction and Illumina Sequencing

After the quality check procedure, RNA with poly-A in eukaryotic total RNA samples was enriched using the TurboCapture 96 mRNA Kit (QIAGEN, Hamburg, Germany). Then, transcriptome sequencing libraries were constructed with the TruSeq RNA Library Preparation Kit V2 (Illumina, San Diego, CA, USA). Briefly, library construction involved capture of mRNA fragmentation, mRNA disruption and synthesis of double-stranded cDNA, end repair, A-tailing, ligation of sequencing adapters, size selection, and library PCR amplification. Qubit 2.0 fluorometer (Life Technologies, Carlsbad, CA, USA) was used to quantified library concentration. Subsequently, the concentration was diluted to 1 ng/µL. The insert size was checked by an Agilent 2100 Bioanalyzer and quantified to greater accuracy using quantitative PCR (q-PCR). The clustering of the index-coded samples was performed on a cBot Cluster Generation System by using TruSeq PE Cluster Kit v3-cBot-HS (Illumina, San Diego, CA, USA) [17]. After cluster generation, the RNA libraries were sequenced on an Illumina HiSeq 2500 platform (Illumina, San Diego, CA, USA). Each treatment was applied in three independent sequencing libraries. The raw sequence data were submitted to NCBI Sequence Read Archive (SRA) (accession number: SUB12082976).

### 2.4. DEG Analysis and Functional Annotation

The raw data generated through high-throughput sequencing were provided in the fastq format sequences. Clean data (clean reads) were obtained by removing reads containing adapter and trimming low-quality bases with Trimmomatic (http://www.usadellab.org/cms/index.php?page=trimmomatic, version 0.39, accessed on 28 May 2022) [18]. Simultaneously, Q20 and GC contents of the clean data were calculated. All the downstream analyses were performed using high-quality clean data. The trimmed mRNA-Seq reads were mapped to the *S. latifolia* reference genome at JGI genome portal (https://mycocosm.jgi.doe.gov/Spalat1/Spalat1.home.html, accessed on 10 May 2022) by using the TopHat software tool (http://ccb.jhu.edu/software/tophat/index.shtml, version 2.1.1, accessed on 28 May 2022) with a default option [19]. Htseq-count (https://pypi.org/project/HTSeq/, version 2.0.2, accessed on 28 May 2022) was used to count the read numbers mapped to each gene. Then, fragments per kilobase of exon per million mapped reads (FPKM) of each gene was used to quantify the abundance of mRNA. The DESeq2 R package (https://bioconductor.org/packages/release/bioc/html/DESeq2.html, version 1.36.0, accessed on 2 June 2022) was used for differential expression analysis of two groups [20]. The Benjamini and Hochberg’s method for controlling the false discovery rate (FDR) was performed to adjust the resulting *p* values. To identify genes responding to different oxalic acid concentrations, a gene with an adjusted *p* value < 0.05 and an absolute value of log2 fold change > 1 was considered as a DEG.

Gene Ontology (GO) enrichment analysis of DGEs was performed using the topGO R package [21]. GO terms with a corrected *p* value of ≤ 0.05 were considered significantly enriched by DEGs. Genome annotation was based on Ensembl and Kyoto Encyclopedia of Genes and Genomes (KEGG) [22]. The statistical enrichment of DEGs in KEGG pathways was performed using ClusterProfiler R package [23].

### 2.5. Quantitative Real-Time PCR (qRT-PCR)-Based Confirmation of Expression Values of Selected Candidate Genes

According to the biological role of genes clarified through GO and KEGG analyses and the levels and differences in gene expression between samples, 6 DEGs were randomly selected for validation through qRT-PCR on a Bio-Rad CFX96 System (Bio-Rad Laboratories, Inc., Hercules, CA, USA). The 18S ribosomal RNA gene (*18S rbs*) was used as the internal control gene. qRT-PCR primers were designed with Primer 6 and are listed in Table 1. The SYBR Premix ExTaq^TM^ kit (Takara, Dalian, China) was used according to the manufacturer’s protocol. Three biological replicates were performed, and the relative RNA expression level was calculated using the 2^−ΔΔCt^ method.

### 2.6. Statistical Analysis

All data are presented as the mean ± standard deviation (SD). Analysis of variance (ANOVA) and Duncan’s multiple range tests (*p* ≤ 0.05) were used to determine the statistically significant difference. The statistical analysis was applied through SPSS version 20.0 software (SPSS Inc., Chicago, IL, USA).

## 3. Results

### 3.1. Mycelial Growth of S. latifolia under Different Oxalic Acid Concentrations

**Figure 1:**
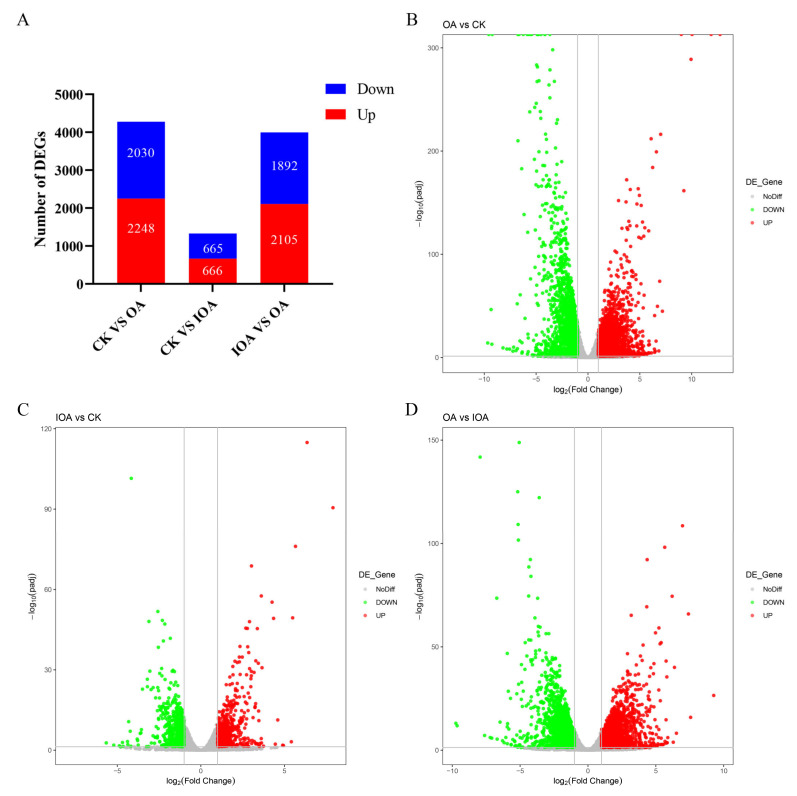
Overview of differential expression analysis for the CK, IOA, and OA treatments. (**A**) Number of up- and down-regulated genes affected by different treatments. (**B**–**D**) Volcano maps of significant DEGs under different oxalic acid concentrations.

After cultivation for 20 days, the *S. latifolia* mycelia grown in PSM containing different oxalic acid concentrations showed different growth characterizations. Under the condition of high oxalic acid concentration (10 mM), *S. latifolia* mycelial growth was significantly inhibited, and the mycelial growth rate was the lowest (Figure 1 and Figure S1). In contrast, the mycelial growth rate significantly increased in PSM medium supplemented with IOA; the growth rate was significantly higher than that with other treatments (Figures S1 and S2). These results further demonstrated that excessive oxalic acid would inhibit *S. latifolia* mycelial growth, and the inhibition of oxalic acid synthesis would significantly promote mycelial growth by reducing the oxalic acid concentration secreted by the mycelia.

### 3.2. Transcriptome Sequencing and Assembly

Over 395 million raw reads and 59.62 GB of raw data were generated from nine samples after excluding low-quality reads (Table S1). Moreover, Q20 values of all cDNA libraries exceeded 95%. After quality filtering, we obtained 46.45, 42.39, and 43.06 million reads for the CK group; 42.94, 39.65, and 41.88 million reads for the IOA group; and 39.65, 43.87, and 38.74 million reads for the OA group (Table S2). Over 88% of the reads were mapped onto the *S. latifolia* genome, with most of them being uniquely mapped (Table S2). Boxplots of the three samples in each group showed similar trends and concentrated range of values (Figure S3). Accordingly, all transcripts of the samples were of high quality and could be used for transcriptional analysis. Furthermore, the gene expression estimation was normalized with the FPKM value, which revealed that 14,521 known genes were expressed in all CK, IOA, and OA mycelium libraries (Table S3).

### 3.3. DEGs and Functional Analysis

To investigate the differential transcription levels among different treatments (CK, IOA, and OA), DEGs were identified and annotated in three comparisons (OA vs. CK, IOA vs. CK, OA vs. IOA), and the normalized read counts (FPKM values) of the nine samples were statistically compared. Compared with the CK group, 4478 DEGs were identified in the OA group (2248 up-regulated and 2030 down-regulated) (Table S4), and 1331 DEGs were identified in the IOA group (666 up-regulated and 665 down-regulated) (Table S5). Furthermore, compared with the IOA group, 3397 DEGs were identified in the OA group (2105 up-regulated and 1892 down-regulated) (Figure 1A, Table S6). Venn diagrams were drawn between the significant DEGs of each comparative combination (Figure S4). The common or unique DEGs of the three combinations are listed in Table S7. The most DEGs were thus observed in the OA group, in which the *S. latifolia* mycelia showed the lowest growth rate, indicating that these DEGs possibly play a crucial role in mycelial growth. Cluster analyses of significant expression under different oxalic acid concentrations were also constructed based on DEGs in different samples (Figure 1B–D). The heatmap of DEGs showed significant difference between groups (Figure S5). Taken together, these results indicated that the oxalic acid concentration affected the expression of many genes, which may be closely related to mycelial growth.

**Figure 2:**
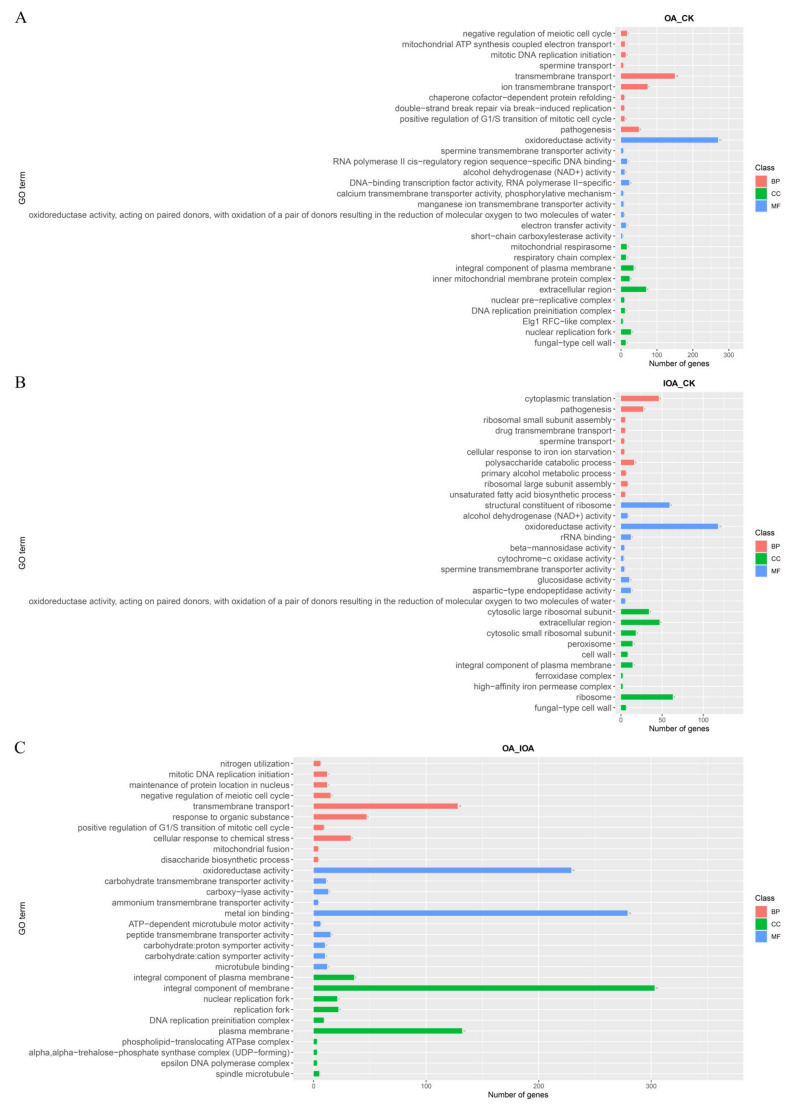
GO database enrichment analysis of DEGs in mycelia between the three treatments. The GO classification map of the (**A**) OA vs. CK, (**B**) IOA vs. CK, and (**C**) OA vs. IOA comparisons.

To investigate the DEGs’ function, the identified genes were annotated using GO and KEGG databases. In the OA vs. CK comparison, 231 terms were enriched from 4478 DEGs, including 56 molecular functions (MFs), 136 biological processes (BPs), and 39 cellular components (CCs) (Figure 2A, Table S8). Among the MFs, the GO enrichment analysis revealed that the significantly down-regulated DEGs were mostly detected to be associated with DNA-binding transcription factor activity (GO:0000981, 17 genes), electron transfer activity (GO:0009055, 13 genes), ubiquitin binding (GO:0043130, 9 genes), alcohol dehydrogenase (NAD+) activity (GO:0004022, 7 genes), and chaperone binding (GO:0051087, 6 genes) (Figure S6A). Among the BPs, the significant DEGs inhibited by high oxalic acid concentrations were mostly detected to be associated with protein folding (GO:0006457, 34 genes), aerobic respiration (GO:0009060, 18 genes), cellular response to heat (GO:0034605, 14 genes), mitochondrial ATP synthesis coupled electron transport (GO:0042776, 10 genes), proton transmembrane transport (GO:1902600, 10 genes), the tricarboxylic acid (TCA) cycle (GO:0006099, 10 genes), and chaperone cofactor-dependent protein refolding (GO:0051085, 8 genes) (Figure S6A). Among the CCs, the significant DEGs inhibited by high oxalic acid concentration were mostly detected to be associated with the integral component of the membrane (GO:0016021, 201 genes), mitochondrial inner membrane (GO:0005747, 59 genes), mitochondrial respirasome (GO:0005746, 15 genes), respiratory chain complex (GO:0098803, 14 genes), COPII−coated ER to Golgi transport vesicle (GO:0030134, 11 genes), mitochondrial respiratory chain complex I (GO:0005747, 6 genes), and mitochondrial respiratory chain complex IV (GO:0005751, 3 genes) (Figure S6A).

In the IOA vs. CK comparison, 117 terms were enriched from 1331 DEGs, including 47 MFs, 55 BPs, and 15 CCs (Figure 2B, Table S9). Among the MFs, the GO enrichment analysis revealed that the significant DEGs influenced by oxalic acid were mostly detected to be associated with oxidoreductase activity (GO:0016491, 57 genes), hydrolase activity—hydrolysis of O-glycosyl compounds (GO:0004553, 25 genes), glucosidase activity (GO:0015926, 9 genes), and alcohol dehydrogenase (NAD+) activity (GO:0004022, 5 genes) (Figure S6B). Among the BPs, the significant DEGs inhibited by high oxalic acid concentrations were mostly detected to be associated with the carbohydrate metabolic process (GO:0005975, 33 genes), polysaccharide catabolic process (GO:0000272, 15 genes), alcohol metabolic process (GO:0006066, 13 genes), carbohydrate transport (GO:0008643, 6 genes), cellulose catabolic process (GO:0030245, 4 genes), and cellular response to reactive oxygen species (ROS) (GO:0034614, 4 genes) (Figure S6B). Among the CCs, the significant DEGs inhibited by high oxalic acid concentrations were mostly detected to be associated with the extracellular region (GO:0005576, 26 genes), peroxisome (GO:0005777, 11 genes), integral component of the plasma membrane (GO:0005887, 9 genes), nucleosome (GO:0000786, 4 genes), cell wall (GO:0005618, 4 genes), and cellular bud neck septin structure (GO:0000399, 2 genes) (Figure S6B).

In the OA vs. IOA comparison, 200 terms were enriched from 3397 DEGs, including 43 MFs, 139 BPs, and 18 CCs (Figure 2C, Table S10). Among the MFs, the GO enrichment analysis revealed that the significant DEGs influenced by oxalic acid were mostly detected to be associated with anion binding (GO:0043168, 215 genes), ribonucleotide binding (GO:0032553, 172 genes), adenyl nucleotide binding (GO:0030554, 145 genes), and transmembrane transporter activity (GO:0022857, 83 genes) (Figure S6C). Among the BPs, the significant DEGs inhibited by high oxalic acid concentrations were mostly detected in protein folding (GO:0006457, 31 genes), mRNA cis splicing via spliceosome (GO:0045292, 25 genes), aerobic respiration (GO:0009060, 15 genes), cellular response to heat (GO:0034605, 14 genes), cellular response to osmotic stress (GO:0071470, 11 genes), iron–sulfur cluster assembly (GO:0016226, 11 genes), chaperone cofactor-dependent protein refolding (GO:0051085, 7 genes), and the trehalose biosynthetic process (GO:0005992, 3 genes) (Figure S6C). Among the CCs, the significant DEGs inhibited by high oxalic acid concentrations were mostly detected to be associated with the integral component of the membrane (GO:0016021, 202 genes), organelle membrane (GO:0031090, 181 genes), spliceosomal complex (GO:0005681, 32 genes), integral component of the plasma membrane (GO:0005887, 22 genes), Golgi apparatus subcompartment (GO:0098791, 11 genes), mitochondrial outer membrane translocase complex (GO:0005742, 4 genes), and the alpha, alpha-trehalose-phosphate synthase complex (UDP-forming) (GO:0005946, 3 genes) (Figure S6C). Given the strong inhibitory effect of high oxalic acid concentrations on *S. latifolia* mycelial growth, we conjecture that the negative effect of high oxalic acid concentrations may be exerted through these processes.

**Figure 3:**
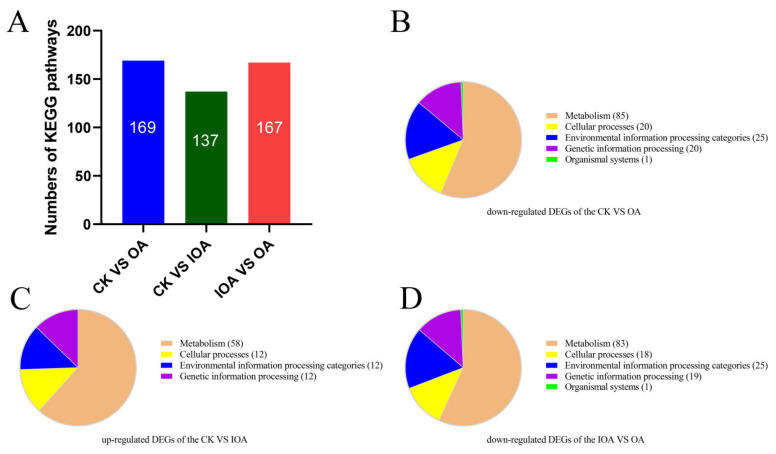
KEGG pathway classification enrichment analysis. (**A**) Number of KEGG pathways enriched for different groups. (**B**) Functional KEGG categories enriched by down-regulated DEGs in the OA vs. CK comparison. (**C**) Functional KEGG categories enriched by up-regulated DEGs in the IOA vs. CK comparison. (**D**) Functional KEGG categories enriched by down-regulated DEGs in the OA vs. IOA comparison.

**Figure 4:**
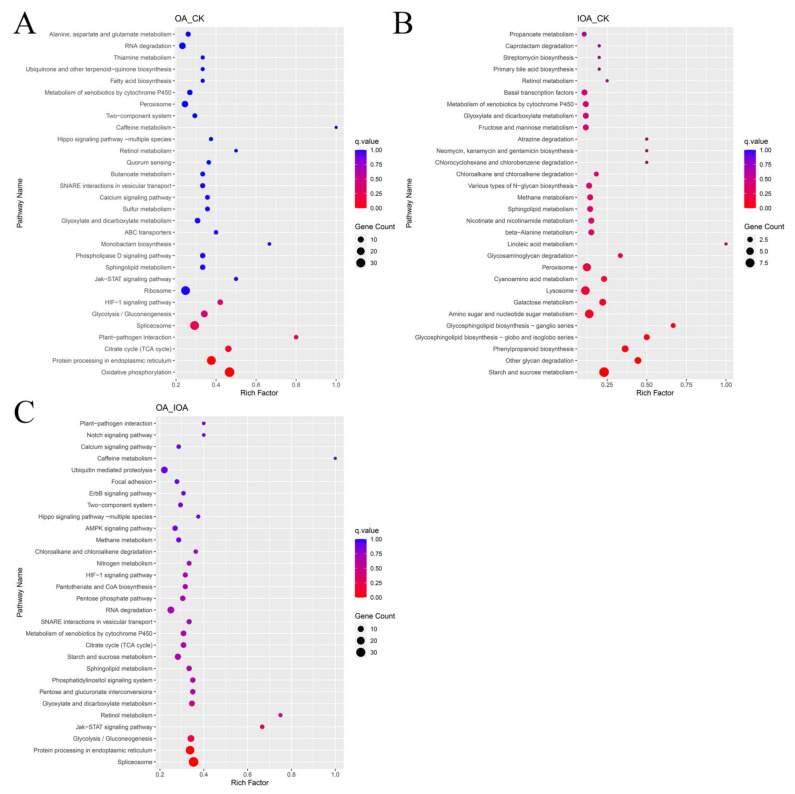
Top 30 enriched KEGG pathways of DEGs in mycelia at different oxalic acid concentrations. (**A**) Enriched KEGG pathways of down-regulated DEGs in the CK and OA groups. (**B**) Enriched KEGG pathways of up-regulated DEGs in the CK and IOA groups. (**C**) Enriched KEGG pathways of down-regulated DEGs in the OA vs. IOA comparison. Richfactor is the ratio of DEGs under the corresponding KEGG term to the total number of DEGs. The larger the richfactor, the greater the degree of enrichment. The q value is the corrected *p* value ranging from 0 to 1, and the circle size indicates the number of enriched genes.

The KEGG pathways with the highest number of DEGs in *S. latifolia* grown in the presence of different oxalic acid concentrations were analyzed. KEGG functional annotation was performed, and the results of KEGG pathway enrichment analysis from the OA vs. CK, IOA vs. CK, and OA vs. IOA comparisons were provided (Figure 3A, Tables S11–S13). In the KEGG enrichment analysis, DEGs influenced by high oxalic acid concentrations were classified into metabolism, genetic information processing, environmental information processing, cellular processes, and organismal systems categories (Figure 3B–D). The top 30 enriched KEGG pathways were identified, which contained significant down-regulated DEGs affected by high oxalic acid concentration in different comparison groups (Figure 4). The high oxalic acid concentrations had the greatest effect on the metabolic category, especially on amino acid metabolism, carbohydrate metabolism, lipid metabolism, metabolism of cofactors and vitamins, xenobiotics biodegradation and metabolism, glycan biosynthesis and metabolism, and energy metabolism. Moreover, many signal transduction pathway genes involved in environmental information processing were also widely inhibited (Tables S14–S16). Accordingly, these pathways are most likely related to the growth of *S. latifolia* mycelia.

### 3.4. DEGs Related to Lignocellulose Degradation Metabolism

Transcripts of genes encoding for lignocellulose-degrading enzymes, including endo-β-1,4-xylanase (mRNA_2483), exo-glucanase (mRNA_8946), glycoside hydrolase (mRNA_449, mRNA_581, mRNA_6544, mRNA_12685, and mRNA_6811), exo-β-1,3-glucosidase (mRNA_6544), and laccases (mRNA_3922), were found in the *S. latifolia* transcriptome (Tables S4–S6). Compared with the IOA group, the expression level of these coding genes was significantly down-regulated in the CK and OA groups (Table 2).

### 3.5. Validation of Transcriptome Data by qRT-PCR

**Figure 5:**
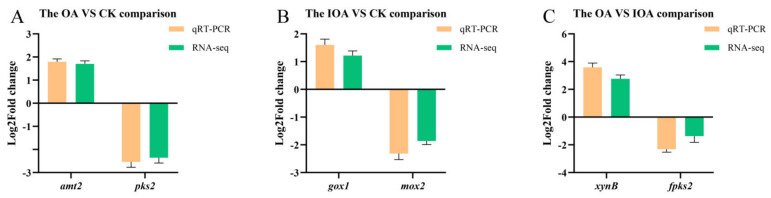
Validation of the DEGs using qRT-PCR. Fold changes of the expression profiles of 6 randomly selected DEGs in 3 comparisons between groups. Each experiment was normalized by the *18S rbs* gene.

qRT-PCR was used to confirm the accuracy of gene expression profiles obtained through RNA-seq. Three up-regulated and three down-regulated DEGs were randomly selected to verify the relative expression levels of RNA-seq data. As shown in Figure 5, the qRT-PCR gene expression patterns were significantly consistent with the RNA-seq data in the three comparisons, which suggested that the RNA-seq data were accurate and reliable.

## 4. Discussion

Oxalic acid is a common low-molecular-weight organic acid commonly produced by the majority of white and brown rot fungi and has been suggested to play multiple roles during the wood degradation process [24]. This organic acid may be involved in both non-enzymatic and enzymatic degradation of lignocellulose for wood decomposition [25]. The oxalic acid concentration was closely related to the wood degradation process, and excessive oxalic acid inhibits the activity of lignocellulose-degrading enzymes [26]. The present work provided comprehensive RNA-seq data that will certainly be fundamental in further investigations of *S. latifolia* mycelial growth, especially for identifying candidate genes associated with slow saprophytic growth caused by high concentrations of oxalic acid.

The most direct effect of oxalic acid on fungal mycelia is its growth rate [27]. In our study, the mycelial growth rate was significantly faster in the IOA group than in the CK and OA groups (Figure S2). Many studies in fungi have profiled transcriptional changes in fungal mycelia in response to oxalic acid [13]. Consistent with our observations, excessive oxalic acid down-regulated the expression level of many genes [14]. The wood-degradation enzyme system of white and brown rot fungi is crucial for the mycelial growth performance and biological efficiency [28]. Many wood-degradation enzymes genes were also significantly down-regulated, such as genes and gene products annotated to cellulase activity (GO:0008810), mannan endo-1,4-beta-mannosidase activity (GO:0016985), and glucosidase activity (GO:0008422) (Figure S6). In particular, the expression levels of many genes related to lignocellulose degradation were significantly down-regulated at high oxalic acid concentrations (Table 2). The degradation ability of lignocellulose was directly related to wood degradation by the mycelium, and will directly affect the growth rate [29]. Furthermore, our previous study also demonstrated that the activities of the lignocellulose-degrading enzyme of *S. latifolia* significantly reduced under high oxalic acid concentration [5]. Therefore, the low degradation efficiency of *S. latifolia* to lignocellulose under high oxalic acid concentration will inevitably reduce its growth rate. Moreover, transcriptomic profiling also revealed that the expression level of many carbohydrate utilization pathways, such as galactose metabolism, starch and sucrose metabolism, and fructose and mannose metabolism, were significantly down-regulated at high oxalic acid concentrations (Table S14, Figure 4). Carbohydrate metabolism plays a special role in carbon/nitrogen exchange, as well as offers metabolic support for fungal mycelial growth [30]. The lower expression level of these carbohydrate metabolic pathways may lead to slower mycelial growth and more decreased biotransformation efficiency. These obtained results may be the reasons for the reduction in the saprophytic growth rate of *S. latifolia* by excessive oxalic acid.

The cell membrane is a thin semi-permeable membrane that plays the function of transmembrane transport of certain substances [31]. Accordingly, as shown in Figure S6C, the expression level of the genes encoding the chemical components of intrinsic cell membrane was significantly down-regulated at high oxalic acid concentrations, which may hinder the transmembrane transport of substances. To maintain the normal transportation ability of the cell membrane, the expression level of energy-metabolism-related genes needs to be up-regulated to complete the active transport processes of certain substances [32]. Energy metabolism of substances is the main vital source of generating energy (ATP) [33]. However, many processes of energy metabolism and pathways were significantly down-regulated at high oxalic acid concentrations, such as the TCA cycle (GO:0006099), the ATP metabolic process (GO:0046034), oxidative phosphorylation (ko00190), pyruvate metabolism (ko00620), and glycolysis/gluconeogenesis (ko00010) (Figure S6A, Table S14). Therefore, these results suggest that high oxalic acid concentrations significantly inhibited the energy production processes and substance absorption by the mycelium, thus affecting the saprophytic growth of the *S. latifolia* mycelium.

Oxidative phosphorylation is a critical coupling reaction wherein energy is released during mitochondria oxidation of substances [34]. Mitochondria are the energy-producing organelles in eukaryotes, mainly through oxidative phosphorylation to generate ATP [35]. In our study, significant DEGs under excessive oxalic acid showed extensive mitochondrial dysfunction (Figure S6A, Table S8). A comparison of transcriptome data also revealed that aerobic respiration was significantly inhibited by excessive oxalic acid (Figure S6C). The succinate dehydrogenase complex (GO:0000104), which is located in the inner mitochondrial membrane and provides electrons for the mitochondrial aerobic and energy-producing respiratory chain of eukaryotic cells, was down-regulated at high oxalic acid concentrations (Table S8) [36]. Mitochondrial dysfunction can also lead to excess production of ROS, thereby causing oxidative stress [37]. However, GO annotation found that the genes encoding ROS scavenging enzymes, such as glutathione peroxidase (GO:0004602) and catalase (GO:0004096) (Tables S9 and S10), were significantly down-regulated at high oxalic acid concentrations [38]. Based on these results, we speculate that mitochondrial dysfunction caused by excessive oxalic acid will reduce energy production, thus inhibiting the growth of *S. latifolia* mycelia.

The fungal mycelium growth is accompanied by continuous synthesis and assembly of cell wall polysaccharides that mainly include glucans, chitin, mannans, and glycoproteins [39]. All living cells are decorated with branched sugar polymers know as glycans. Glycan biosynthesis is directly related to the mycelial growth of filamentous fungi [40]. In our study, transcriptome analysis found that the expression level of many genes involved in glycan synthesis and metabolism were significantly down-regulated, such as N-glycan biosynthesis (ko00513), glycosphingolipid biosynthesis (ko00603), mannose type O-glycan biosynthesis (ko00515), and steroid biosynthesis (ko00100) (Tables S13–S15). The slow saprophytic growth of *S. latifolia* mycelia may be due to the decreased ability of the fungus to synthesize glycans at high oxalic acid concentrations.

Mycelial growth is also accompanied by the synthesis of numerous proteins and their assembly and post-translational modifications. Ribosomes are ribonucleoprotein particles, and act as molecular machinery for protein synthesis in the cells [41,42]. Ribosomes also played important roles in DNA repair, development, and cell division [43,44]. However, genes encoding DNA replication proteins and ribosome proteins were strongly inhibited at high oxalic acid concentrations (Figure 4A and Figure S6A), which suggested that ribosome and DNA replication activities are required for *S. latifolia* mycelial growth. Furthermore, many genes associated with amino acid metabolism and vitamins were enriched in the group of down-regulated DEGs at high oxalic acid concentrations, including the synthesis and catabolism of various amino acids (Tables S14–S16). Amino acid metabolism and vitamin metabolism are crucial players in fungal mycelia growth and fruiting body formation, and are involved in the synthesis of tissue proteins, many ammonia-containing substances, and enzyme-catalyzed reaction processes [45,46]. The significant enrichment analysis reveals that the biological pathways probably relate to saprophytic growth of *S. latifolia* mycelia. However, the mechanism of biosynthesis regulated by excessive oxalic acid needs to be revealed by in-depth research.

Previous studies have reported that many signal transduction pathways are involved in fungal mycelial growth, such as cAMP/PKA pathways [47], MAPK signal pathways [48], the PI3K–Akt signaling pathway [49], and the mTOR signaling pathway [50]. Excessive oxalic acid can cause osmotic stress for the fungal mycelium, and this signal is transmitted to activate the expression of some signal transduction pathways [51]. In the case of MAPK pathways, it is helpful for fungi to adapt to various environments and maintain the normal growth of mycelia. Our transcriptome analysis and gene expression profiles in comparison among groups revealed that the expression levels of multiple genes in these signal pathways were significantly down-regulated by excessive oxalic acid, thereby impairing the expression of these pathways and inhibiting the saprophytic growth of *S. latifolia* mycelia. Moreover, our previous study also revealed that the MAPK, PI3K–Akt, and mTOR signaling pathways play important roles in primordium formation and fruiting body development [52]. Therefore, many down-regulated genes in these signal pathways will seriously restrict the mycelial growth and the fruiting body development of the *S. latifolia*, leading to the prolongation of the cultivation cycle.

## 5. Conclusions

Our work presents the first of comparative transcriptome analysis of *S. latifolia* growing in PSM with different oxalic acid concentrations. In summary, the comparison of gene expression profiles showed that high oxalic acid concentrations inhibited many *S. latifolia* pathways related to the growth and development of mycelia, such as lignocellulose degradation metabolism, carbohydrate metabolism, and signal transduction processes. The inhibition of these metabolic pathways may be the reason for the slow saprophytic growth and long cultivation cycle of *S. latifolia*. On the one hand, observed differential expression patterns of DEGs suggest a seemingly complex regulation of oxalic acid in *S. latifolia*. On the other hand, transcriptome analysis identified some valuable candidate genes for further studies. However, the mechanism of oxalic acid in the degradation of pine sawdust by *S. latifolia* must be complicated and needs to be further explored. In conclusion, our results reveal the new mechanisms underlying the inhibitory effect of excessive oxalic acid on *S. latifolia* mycelia. Results of the present work will be beneficial for future work seeking to investigate in depth the initiation of saprophytic growth of *S. latifolia*.

## Figures and Tables

**Table 1:** The list of primers for qRT-PCR validation.

Gene	Description	Primer Sequences (5′-3′)
*18S rbs*	18S rRNA gene	Forward: GCGCTACACTGACAGAGCCA
		Reverse: GCGGTGTGTACAAAGGGCAG
*mRNA_12253*	*pks2*; Polyketide synthase	Forward: GTTCTTGATTGATCGCGGGC
		Reverse: TGGATTTCTGCGAGGCCATT
*mRNA_9796*	*amt2*; Aminotransferase	Forward: CTTCTTTGGCAACCTGCACC
		Reverse: CGTATCGAAATTCCGCGAGC
*mRNA_9500*	*mox2*; Monooxygenase	Forward: GCTTCACATCGAGTTCCCCA
		Reverse: AAGCAACTCCTTCCCAACCC
*mRNA_5483*	*gox1*; Glycolate oxidase	Forward: GACGATCACATCCTCGGCTT
		Reverse: ATATCGTCTCCGTCCCGTCT
*mRNA_5970*	*fks2*; 1,3-beta-Glucan synthase	Forward: TGTCGCACTCCATCTCATCG
		Reverse: AAGCGATCGTTTGGACACCT
*mRNA_7010*	*xynB*; Glycoside hydrolase	Forward: GACCGTACAGTCTGGCTCTG
		Reverse: ATGGTACGGGTCTGTAGGCT

**Table 2:** Down-regulated DEGs enriched in wood-degrading enzymes in the presence of high oxalic acid concentration.

Gene ID	Log_2_ Fold Change	*p* Value	Function Description	Gene Name	GO ID	GO Term
mRNA_2483	1.52 × 10^−34^	1.93 × 10^−33^	Endo-β-1,4-xylanase	*xyl1*	GO:0005576	Endo-1,4-β-xylanase activity
mRNA_8946	−1.637572757	7.47 × 10^−5^	Exo-glucanase	*GH12*	GO:0008810	Cellulase activity
mRNA_449	−2.254476744	1.58 × 10^−2^	Glycoside hydrolase	*GH12*	GO:0008810	Cellulase activity
mRNA_581	2.118043022	2.87 × 10^−10^	Glycoside hydrolase	*GH5*	GO:0008810	Cellulase activity
mRNA_12685	−1.591430674	8.83 × 10^−42^	Glycoside hydrolase	*GH16*	GO:0015926	Glucosidase activity
mRNA_6811	−1.034962051	2.07 × 10^−6^	Glycoside hydrolase	*GH71*	GO:0051118	Glucan endo-1,3-alpha-glucosidase activity
mRNA_6544	−1.398229479	2.08 × 10^−54^	Exo-β-1,3-glucosidase	*EXG*	GO:0004338	Glucan exo-1,3-beta-glucosidase activity
mRNA_3922	−1.297733133	1.18 × 10^−6^	Laccase	Lac	GO:0046274	Lignin catabolic process

## Data Availability

All data presented in this study are available within this article.

## Supplementary Materials

The following supporting information can be downloaded at: https://www.mdpi.com/article/10.3390/cells11223636/s1, Figure S1: Effect of oxalic acid on the growth of *S. latifolia* mycelia in PSM medium. (A) PSM medium supplemented with IOA; (B) PSM medium; (C) PSM medium supplemented with OA; Figure S2: Growth rate of the *S. latifolia* mycelium at different oxalic acid concentrations. IOA: PSM supplemented with 4 mM 3,3-difluorooxaloacetate, CK: PSM, OA: PSM supplemented with 10 mM oxalic acid. Data were analyzed using Duncan’s ANOVA test. Error bars represent the standard deviation of three replicates. Different letters indicate significant differences between the lines (*p* ≤ 0.05); Figure S3: Transcriptional relationships among the nine samples; Figure S4: Venn diagram of significantly DEGs. OA_CK means OA VS CK, IOA_CK means IOA VS CK, and OA_IOA means OA VS IOA; Figure S5: The heatmap of significantly DEGs at different oxalic acid concentrations; Figure S6: GO functional annotation of significant DEGs at different oxalic acid concentrations. The GO classification map of the (A) OA VS CK, (B) IOA VS CK, (C) OA VS IOA comparisons. OA_CK means OA VS CK, IOA_CK means IOA VS CK, and OA_IOA means OA VS IOA. The most significant 30 enrichment terms were selected from the GO enrichment analysis results to draw the histogram; Table S1: Summary of the sequencing and assembly; Table S2: Mapped results of the RNA sequencing data; Table S3: Summary of the transcriptome annotation and gene expression data; Table S4: Clustering analysis of significant DEGs in the OA VS CK comparison; Table S5: Clustering analysis of significant DEGs in the IOA VS CK comparison; Table S6: Clustering analysis of significant DEGs in the OA VS IOA comparison; Table S7: The common or unique DEGs of the three comparison; Table S8: GO enrichment analysis of the DEGs in the OA VS CK comparison; Table S9: GO enrichment analysis of the DEGs in the IOA VS CK comparison; Table S10: GO enrichment analysis of the DEGs in the OA VS IOA comparison; Table S11: The functional categories of the KEGG pathway enriched for the OA VS CK comparison; Table S12: The functional categories of the KEGG pathway enriched for the IOA VS CK comparison; Table S13: The functional categories of the KEGG pathway enriched for the OA VS IOA comparison; Table S14: KEGG pathway classification enrichment analysis of significant down-regulated DEGs in the OA VS CK comparison; Table S15: KEGG pathway classification enrichment analysis of significant up-regulated DEGs in the IOA VS CK comparison; Table S16: KEGG pathway classification enrichment analysis of significant down-regulated DEGs in the OA VS IOA comparison.

## Abbreviations

PDPA, potato dextrose peptone agar; PSM, pine sawdust medium; CK, PSM; OA, PSM with 10 mM oxalic acid; IOA, PSM with 4 mM 3,3-difluorooxaloacetate; FPKM, fragments per kilobase of exon per million mapped reads; DEGs, differentially expressed genes; FDR, false discovery rate; GO, Gene Ontology; KEGG, Kyoto Encyclopedia of Genes and Genomes; qRT-PCR, quantitative real-time PCR.
