# Supplementary material for: Transcriptome Analysis Revealed the Mechanism of Inhibition of Saprophytic Growth of Sparassis latifolia by Excessive Oxalic Acid
Source: Cells. 2022 Nov 16;11(22):3636. doi: 10.3390/cells11223636 (PMC9688073; doi:10.3390/cells11223636)
Supplement: Supplementary file 1 [file cells-11-03636-s001.zip › Supplementary file.pdf]

Transcriptome analysis revealed the mechanism of inhibition of saprophytic growth of *Sparassis latifolia* by excessive oxalic acid

Zhiheng Qiu [1,2†], Xinyi Wang [1,2†], Shuang Wang [1,2], Nuo Cai [1,2], Jing Huang [1,2], Miaoyue Wang [1,2], Lili Shu [1,2*] and Tianlai Li [1,2*]

1 College of Horticulture, Shenyang Agricultural University, Shenyang 110866, China
2 Key Laboratory of Protected Horticulture of Education Ministry and Liaoning Province, Shenyang 110866, China.
* Correspondence: shulili1986@syau.edu.cn (L.S.); 1982500005@syau.edu.cn (T.L.); Tel.: +86-024-88487143 (L.S.)
† These authors contribute equally to this work.

Figure S1. Effect of oxalic acid on the growth of S. latifolia mycelia in PSM medium. **(A)** PSM medium supplemented with IOA; **(B)** PSM medium; **(C)** PSM medium supplemented with OA.

Figure S2. Growth rate of the *S. latifolia* mycelium at different oxalic acid concentrations. IOA: PSM supplemented with 4 mM 3,3-difluorooxaloacetate, CK: PSM, OA: PSM supplemented with 10 mM oxalic acid. Data were analyzed using Duncan's ANOVA test. Error bars represent the standard deviation of three replicates. Different letters indicate significant differences between the lines ($P \leq 0.05$).

Figure S3. Transcriptional relationships among the nine samples.

Figure S4. Venn diagram of significantly DEGs. OA_CK means OA VS CK, IOA_CK means IOA VS CK, and OA_IOA means OA VS IOA.

Figure S5. The heatmap of significantly DEGs at different oxalic acid concentrations.

Figure S6. GO functional annotation of significant DEGs at different oxalic acid concentrations. The GO classification map of the **(A)** OA VS CK, **(B)** IOA VS CK, **(C)** OA VS IOA comparisons. OA_CK means OA VS CK, IOA_CK means IOA VS CK, and OA_IOA means OA VS IOA. The most significant 30 enrichment terms were selected from the GO enrichment analysis results to draw the histogram.

Table S1. Summary of the sequencing and assembly.

Sample	Reads length (bp)	Raw reads	Raw data (G)	Q20 (%)	GC (%)
CK1	150	48,181,698	7.25	98.16	56.4
CK2	150	44,076,784	6.65	98.10	55.98
CK3	150	44,590,446	6.73	98.25	56.14
IOA1	150	44,961,636	6.79	97.77	56.12
IOA2	150	43,253,478	6.53	96.43	55.53
IOA3	150	43,572,276	6.58	97.97	56.08
OA1	150	41,359,266	6.20	97.66	55.88
OA2	150	45,637,988	6.85	97.83	55.97
OA3	150	40,287,688	6.04	97.79	56.23

Table S2. Mapped results of the RNA sequencing data.

Sampl e	Total reads after filtered	Mapped on reference		Multiple mapped		Uniquely Mapped	
		Reads	%	Reads	%	Reads	%
CK1	46,454,464	41,996,785	90.4	767,182	1.65	41,229,603	98.35
CK2	42,387,366	38,059,277	89.79	586,887	1.38	37,472,390	98.62
CK3	43,062,712	38,863,148	90.25	596,485	1.39	38,266,663	98.61
IOA1	42,940,772	38,941,480	90.69	620,741	1.45	38,320,739	98.55
IOA2	39,613,248	34,891,308	88.08	551,542	1.39	34,339,766	98.61
IOA3	41,879,110	37,974,499	90.68	581,046	1.39	37,393,453	98.61
OA1	39,650,740	35,704,588	90.05	480,067	1.21	35,224,521	98.79
OA2	43,886,300	39,503,793	90.01	622,995	1.42	38,880,798	98.58
OA3	38,737,172	34,942,552	90.2	576,243	1.49	34,366,309	98.51
