# Supplementary figures and images for: Transcriptome Analysis Revealed the Mechanism of Inhibition of Saprophytic Growth of Sparassis latifolia by Excessive Oxalic Acid
Source: Cells. 2022 Nov 16;11(22):3636. doi: 10.3390/cells11223636 (PMC9688073; doi:10.3390/cells11223636)

A

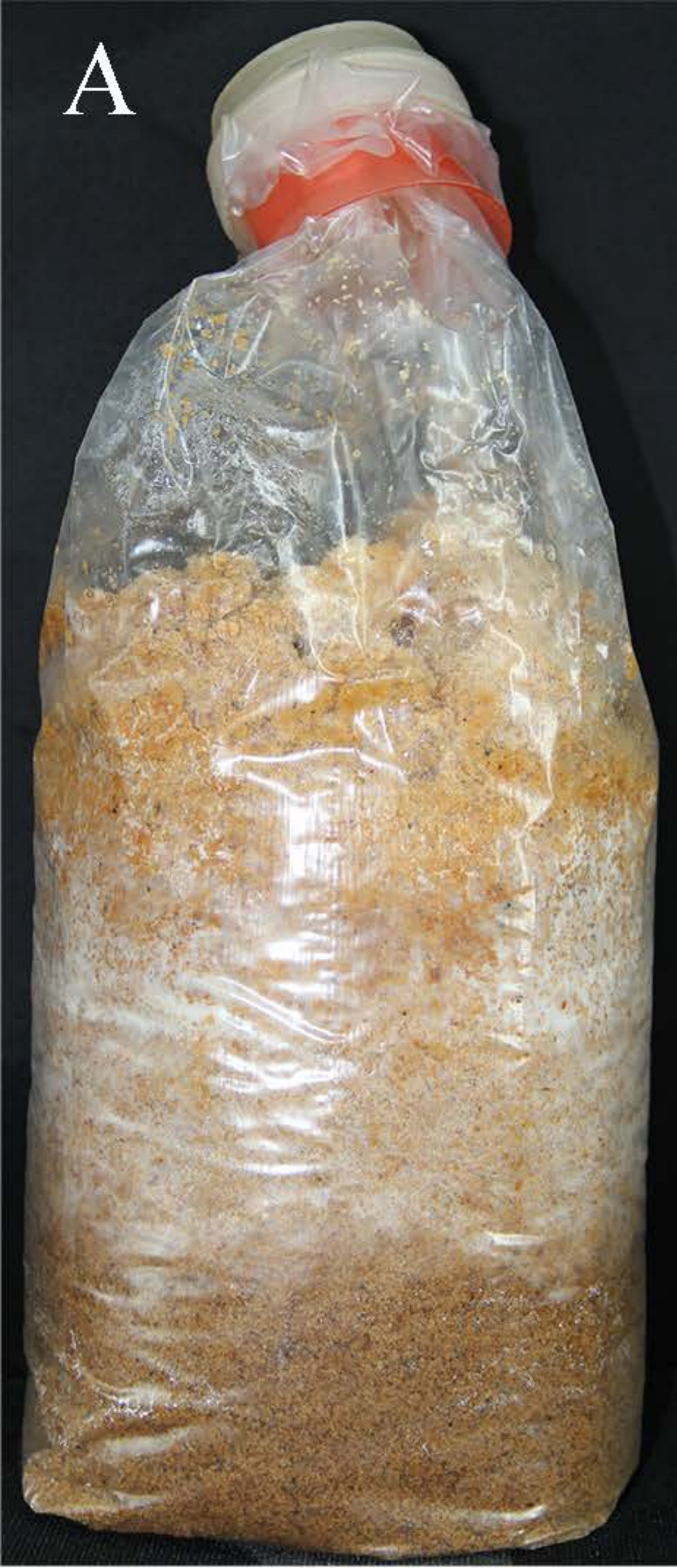

B

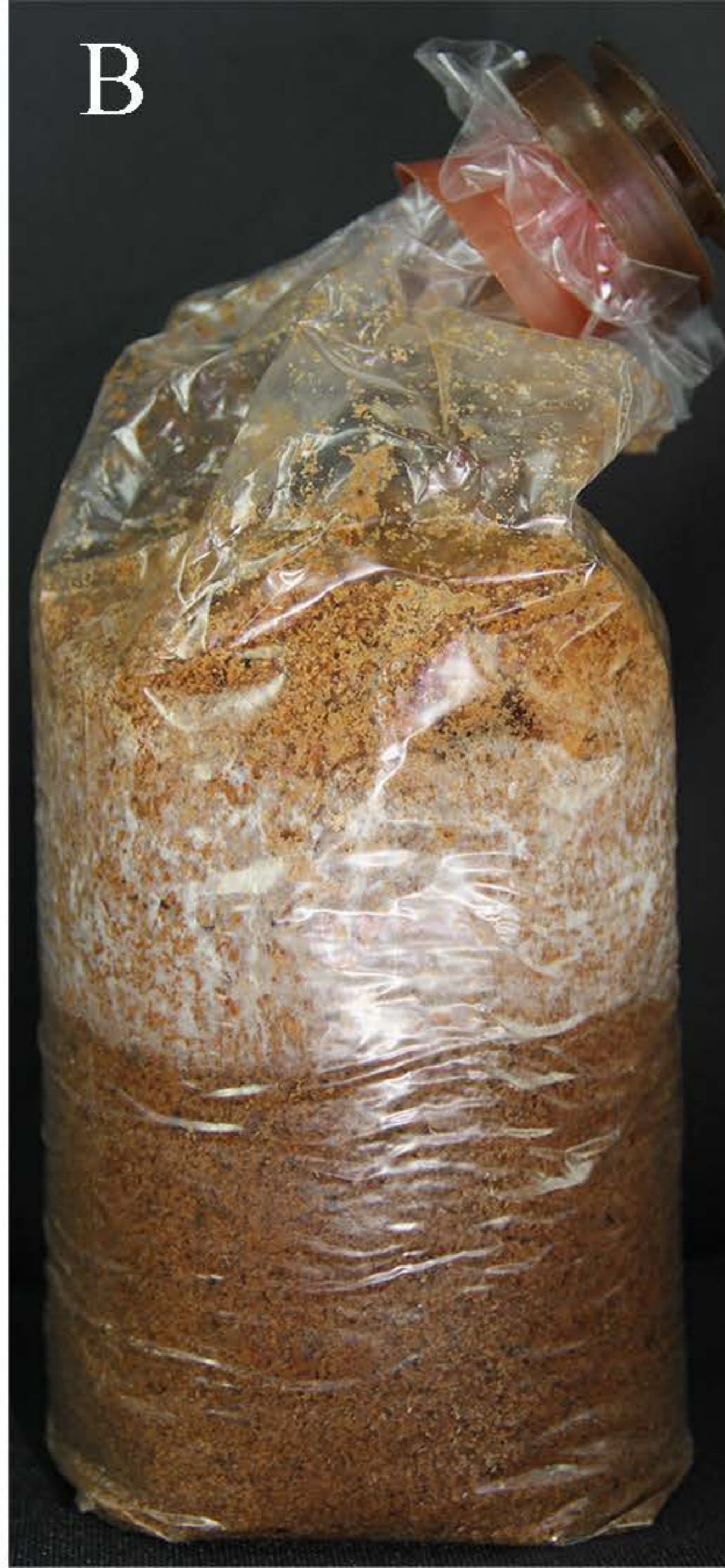

C

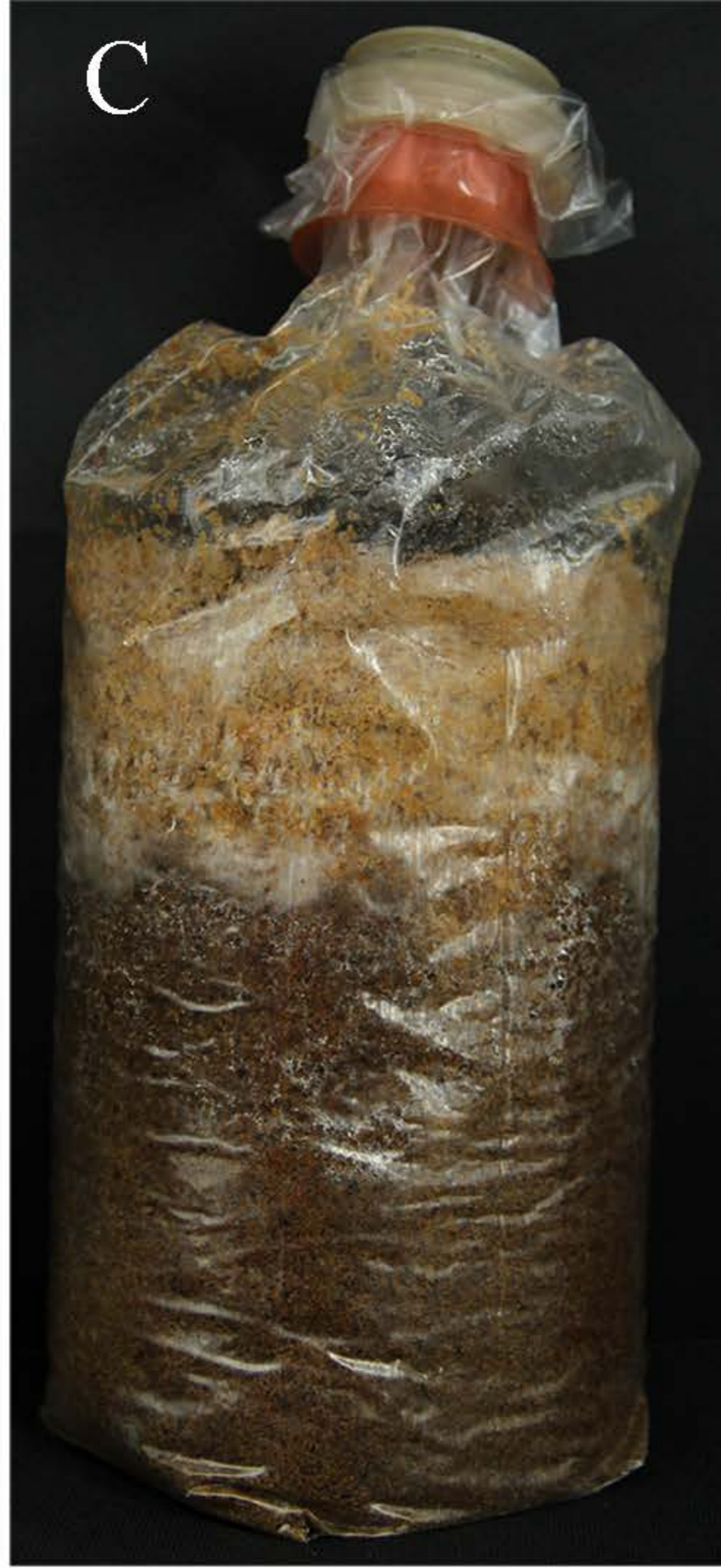

Supplement: Supplementary file 1 [file cells-11-03636-s001.zip › Figure S1.pdf]

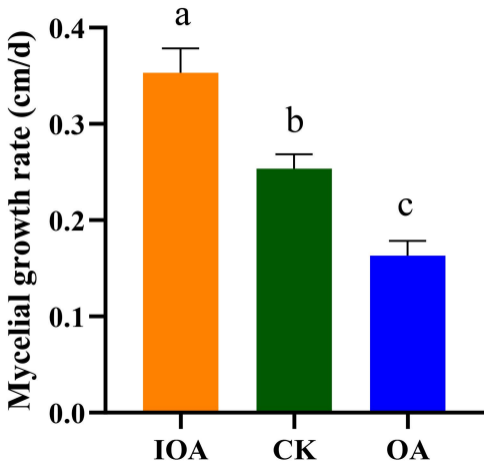

Supplement: Supplementary file 1 [file cells-11-03636-s001.zip › Figure S2.pdf]

Boxplot of FPKM Distribution

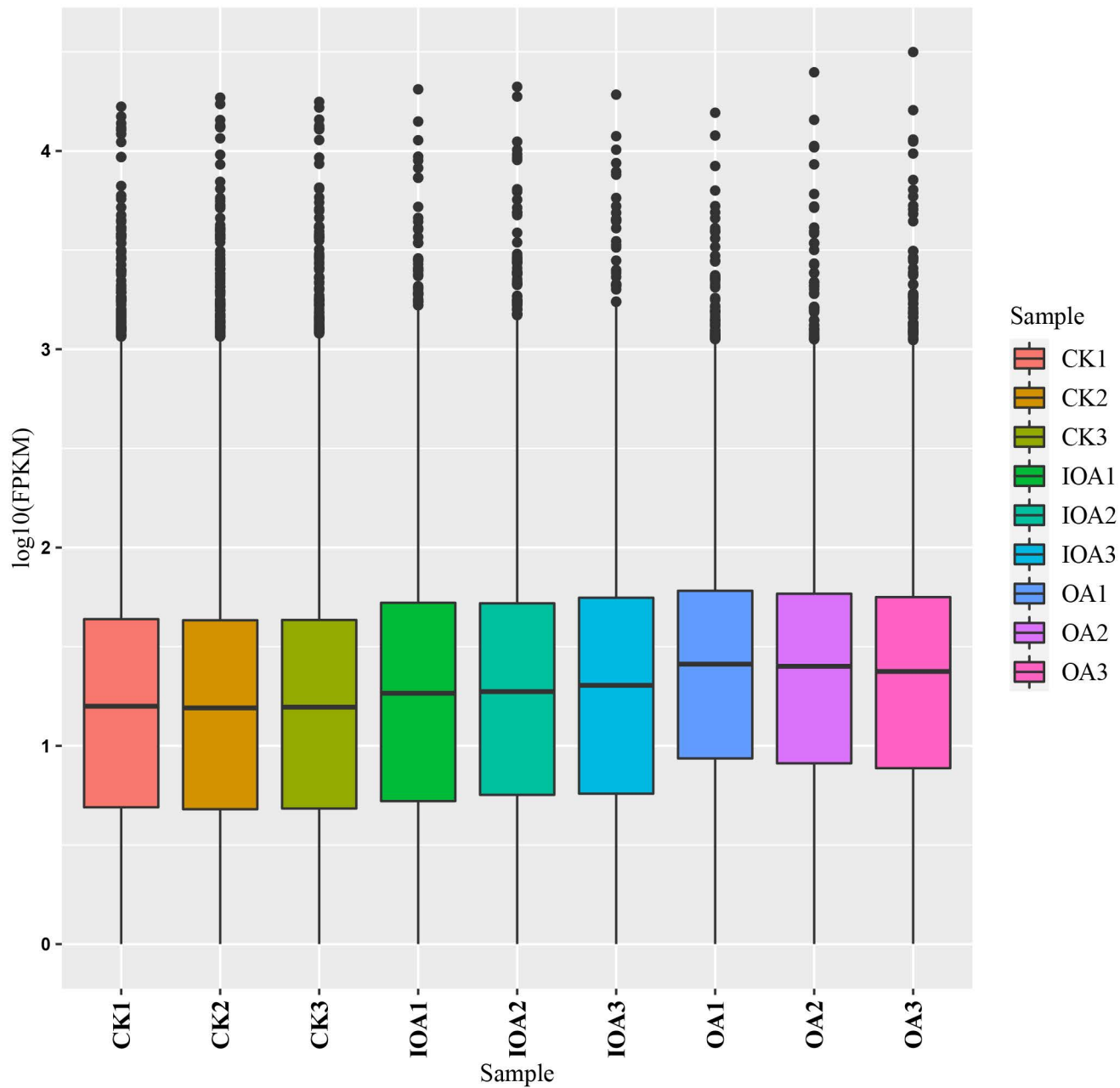

Supplement: Supplementary file 1 [file cells-11-03636-s001.zip › Figure S3.pdf]

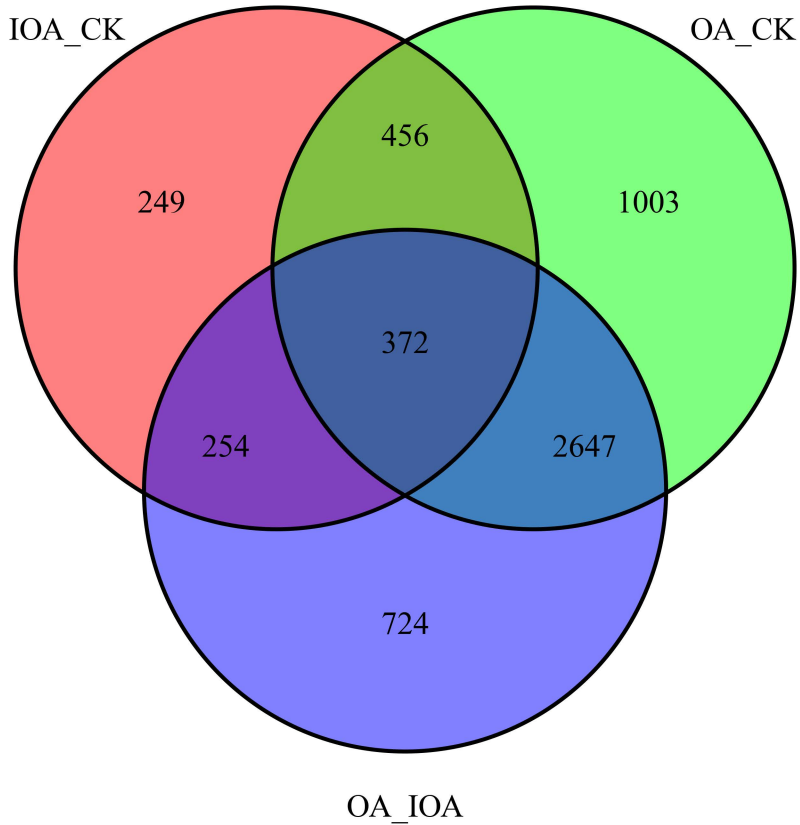

Supplement: Supplementary file 1 [file cells-11-03636-s001.zip › Figure S4.pdf]

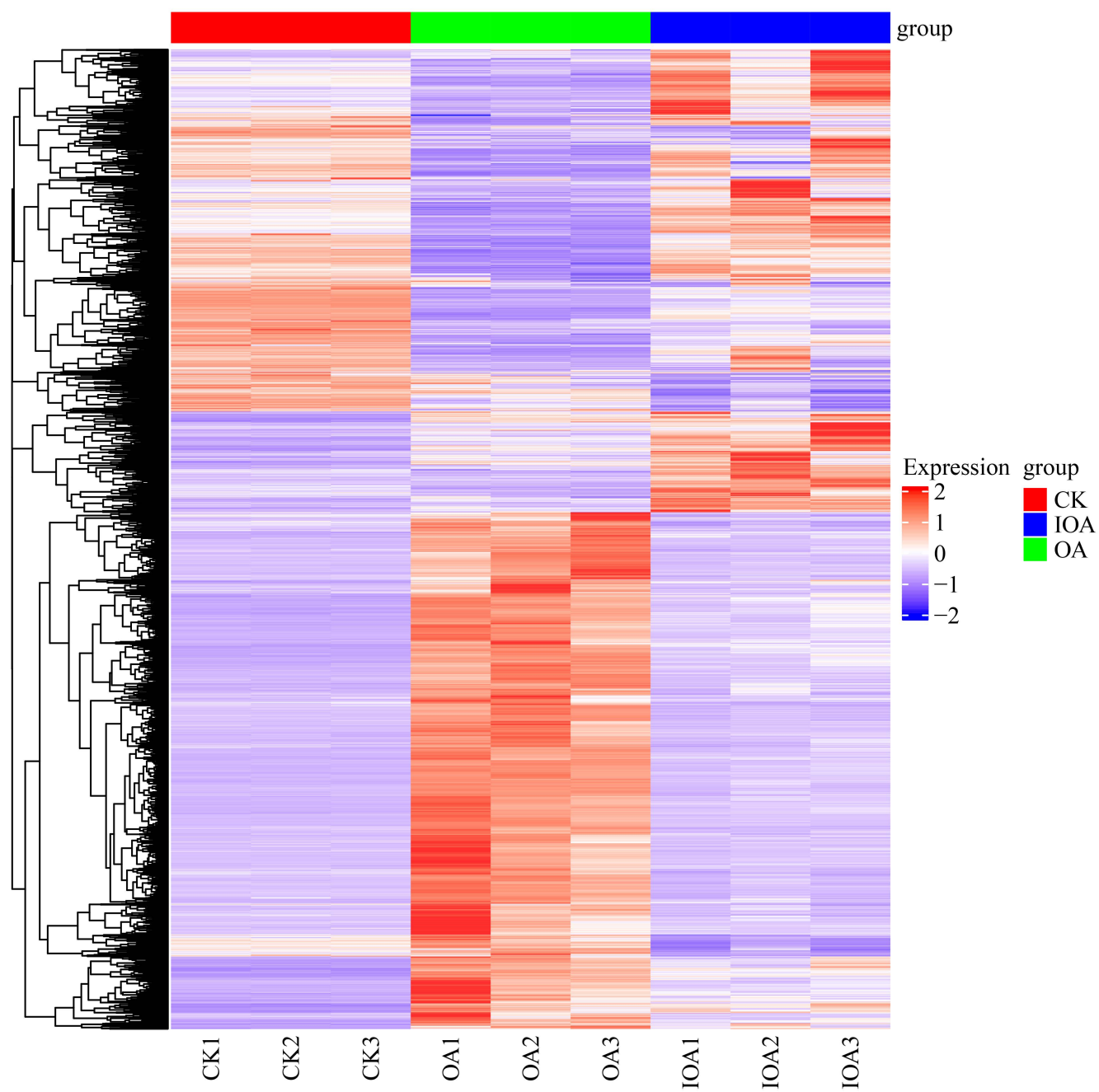

Supplement: Supplementary file 1 [file cells-11-03636-s001.zip › Figure S5.pdf]

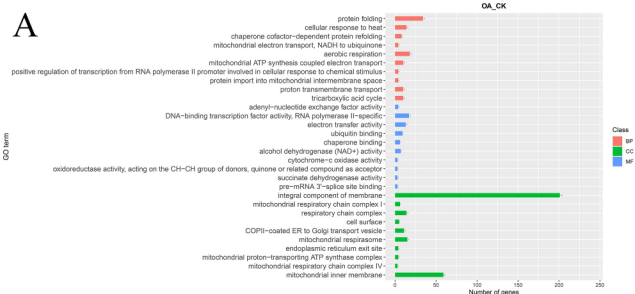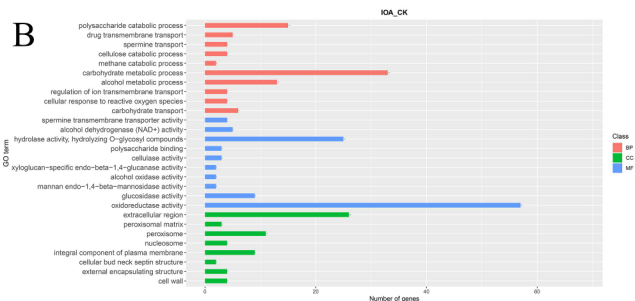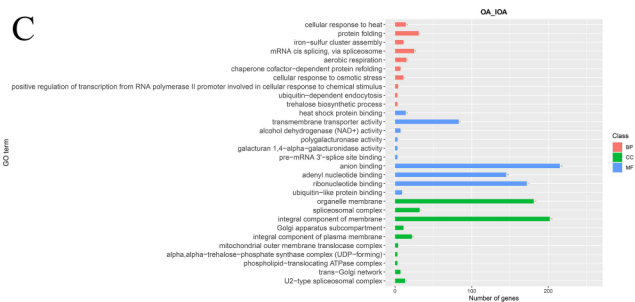

Supplement: Supplementary file 1 [file cells-11-03636-s001.zip › Figure S6.pdf]
